# Supplementary material for: Multi-Observer Study on Diagnostic Accuracy of Pediatric Renal Tumors Imaged with Higher-Harmonic-Generation Microscopy
Source: Cancers (Basel). 2025 May 18;17(10):1693. doi: 10.3390/cancers17101693 (PMC12109953; doi:10.3390/cancers17101693)

Supplementary Data

S1: Overview of the question sheet

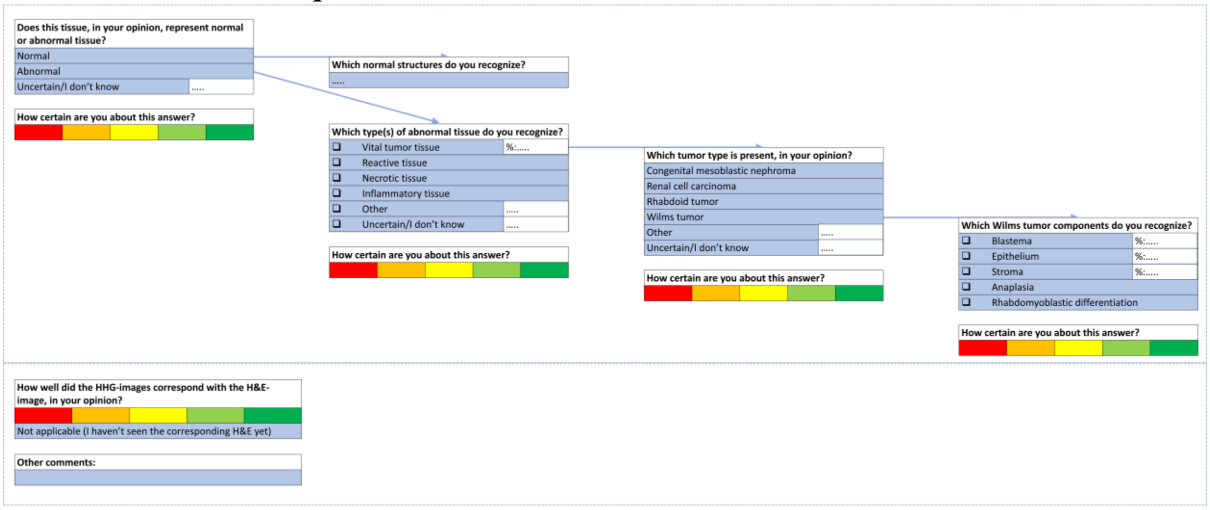

Supplementary Data 1 Overview of the questions that were asked in the Slide Score study. Most questions were dependent on the previous answer, which is visualized by arrows. These questions only appeared if a specific answer was given, to make sure that only the relevant questions were asked. Text fields represent questions where explanations or percentages could be given. Checkboxes represent questions where multiple answers could be selected. Most questions were obligatory, only the checkbox options and 'Other comments' were not obligatory. After each multiple-choice question, the uncertainty was given by very uncertain (red), uncertain (orange), neutral (yellow), certain (yellow green), very certain (green). Following the HHGM image, the pathologists received the corresponding H&E section with the same question sheet and could not see nor change their answers of the previous. In addition, pathologists assessed the correspondence between the HHGM images and the H&E image, using the following categories: very bad (red), bad (orange), neutral (yellow), good (yellow green) and very good (green). Since it was not possible to make separate question sheets for HHGM and H&E cases, the option `Not applicable, I haven't seen the corresponding histology yet' was added to these options.

S2: Overview of the cases

Supplementary Data 2 Overview of the cases that were included in this study. To include normal renal tissue in this study as well, six samples were taken from normal renal tissue sufficiently far away from the tumor. When two samples from the same patient were present, one of the samples was moved to the end of the Slide Score study.

**Case**	**Organ**	**Patient**	**Diagnosis**
01	Kidney	Patient 1	Normal kidney reference
02	Kidney	Patient 2	Wilms tumor
03	Lung	Patient 3	Wilms tumor metastasis
04	Kidney	Patient 4	Wilms tumor
05	Kidney	Patient 5	Wilms tumor
06	Kidney	Patient 6	Wilms tumor
07	Lymph node	Patient 7	Renal cell carcinoma metastasis
08	Kidney	Patient 8	Wilms tumor
09	Kidney	Patient 9	Normal kidney reference
10	Kidney	Patient 10	Wilms tumor
11	Kidney	Patient 11	Wilms tumor
12	Kidney	Patient 12	Wilms tumor
13	Kidney	Patient 13	Wilms tumor
14	Kidney	Patient 14	Normal kidney reference
15	Kidney	Patient 15	Wilms tumor
16	Kidney	Patient 16	Wilms tumor
17	Kidney	Patient 17	Wilms tumor
18	Kidney	Patient 18	Wilms tumor
19	Kidney	Patient 1	Wilms tumor
20	Kidney	Patient 4	Wilms tumor
21	Kidney	Patient 5	Wilms tumor
22	Kidney	Patient 7	Renal cell carcinoma
23	Kidney	Patient 9	Congenital mesoblastic nephroma
24	Kidney	Patient 13	Normal kidney reference
25	Kidney	Patient 14	Wilms tumor
26	Kidney	Patient 15	Normal kidney reference
27	Kidney	Patient 16	Normal kidney reference
28	Kidney	Patient 17	Normal kidney reference
29	Kidney	Patient 18	Wilms tumor

S3: Pathologists' assessment of normal and abnormal tissue

Supplementary Data 3 Assessment of normal (N) and abnormal tissue (A). Colors are used for a better visualization: normal (green), abnormal (orange) and uncertain (white). The color of the 'case' column represents the macroscopic evaluation, i.e. whether the tissue was provided as tumor sample (red) or normal sample (green). Each case consists of two rows, an HHG part (upper row) and an H&E part (lower row). The columns P1-P10 represent the ten pathologists in random order. The consensus was determined as the majority opinion (≥ 6 pathologists in agreement). In one case there was no clear majority opinion (case 09 H&E) and the consensus was classified as abnormal (orange). The consensus was used to calculate the diagnostic characteristics for the distinction between normal and abnormal tissue.

Case		Pathologists										Consensus
		P1	P2	P3	P4	P5	P6	P7	P8	P9	P10	
01 N	HHG	A	N	N	N	N	N	N	N	N	N	
	H&E	N	N	N	N	N	N	N	N	N	N	
02 WT	HHG	A	A	A	A	A	A	A	A	A	A	
	H&E	A	A	A	A	A	A	A	A	A	A	
03 WT	HHG	A	A	A	A	A	?	A	A	A	A	
	H&E	A	A	A	A	A	A	A	A	A	A	
04 WT	HHG	A	A	A	A	A	A	A	A	A	A	
	H&E	A	A	A	A	A	A	A	A	A	A	
05 WT	HHG	A	A	A	A	A	A	A	A	A	A	
	H&E	A	A	A	A	A	A	A	A	A	A	
06 WT	HHG	A	A	A	A	A	A	A	A	A	A	
	H&E	A	A	A	A	A	A	A	A	A	A	
07 RCC	HHG	A	A	A	N	A	A	A	A	A	A	
	H&E	A	A	A	A	A	A	A	A	A	A	
08 WT	HHG	A	A	A	A	A	A	A	A	A	A	
	H&E	A	A	A	A	A	A	A	A	A	A	
09 N	HHG	?	A	A	N	A	N	A	A	A	N	
	H&E	N	A	A	N	A	A	N	A	N	N	
10 WT	HHG	A	A	A	A	A	A	A	A	A	A	
	H&E	A	A	A	A	A	A	A	A	A	A	
11 WT	HHG	A	A	A	A	A	A	A	A	A	A	
	H&E	A	A	A	A	A	A	A	A	A	A	
12 WT	HHG	A	A	A	A	A	?	A	A	A	A	
	H&E	A	A	A	A	A	A	A	A	A	A	
13 WT	HHG	A	A	A	A	A	A	A	A	A	A	
	H&E	A	A	A	A	A	A	A	A	A	A	
14 N	HHG	?	N	N	N	N	N	N	N	N	N	
	H&E	N	N	N	N	N	N	N	N	N	N	
15 WT	HHG	A	A	A	A	A	A	A	A	A	A	
	H&E	A	A	A	A	A	A	A	A	A	A	
16 WT	HHG	A	A	A	A	A	?	A	A	A	?	
	H&E	A	A	A	A	A	A	A	A	A	?	
17 WT	HHG	A	A	A	A	A	A	A	A	A	A	
	H&E	A	A	A	A	A	A	A	A	A	A	
18 WT	HHG	A	A	A	A	A	A	A	A	A	A	
	H&E	A	A	A	A	A	A	A	A	A	A	
19 WT	HHG	A	A	A	A	A	A	A	A	A	A	
	H&E	A	A	A	A	A	A	A	A	A	A	
20 WT	HHG	A	A	A	A	A	A	A	A	A	A	
	H&E	A	A	A	A	A	A	A	A	A	A	
21 WT	HHG	A	A	A	A	A	A	A	A	A	A	
	H&E	A	A	A	A	A	A	A	A	A	A	
22 RCC	HHG	A	A	A	A	A	A	A	A	A	A	
	H&E	A	A	A	A	A	A	A	A	A	A	
23 CMN	HHG	A	A	A	A	A	A	A	A	A	A	
	H&E	A	A	A	A	A	A	A	A	A	A	
24 N	HHG	N	N	N	N	N	N	N	N	N	N	
	H&E	N	N	N	N	N	N	N	N	N	N	
25 WT	HHG	A	A	A	A	A	A	A	A	A	A	
	H&E	A	A	A	A	A	A	A	A	A	A	
26 N	HHG	?	N	A	N	N	N	?	N	N	N	
	H&E	N	A	N	N	N	N	N	N	N	N	
27 N	HHG	N	N	N	N	N	N	A	N	N	N	
	H&E	N	A	N	N	N	N	A	N	N	N	
28 N	HHG	N	N	N	N	A	N	A	N	N	N	
	H&E	N	N	N	N	N	N	N	N	N	N	
29 WT	HHG	A	A	A	A	A	A	A	A	A	A	
	H&E	A	A	A	A	A	A	A	A	A	A	

Legenda	
N	Normal
A	Abnormal
?	Uncertain

S4: Statistical analysis of normal and abnormal tissue

Supplementary Data 4 Statistical analysis (sensitivity, specificity, PPV, NPV, overall agreement and Cohen's kappa) based on the HHGM assessment of each pathologist compared to their own H&E assessment, regarding normal versus abnormal. Calculations are given in parenthesis, 95% confidence intervals are given in square brackets. Colors are used for a better visualization of the percentages: ●0%-24%, ●25%-49%, ●50%-74%, ●75%-100%, and for the kappa values: <0.00, 0.00-0.19, 0.20-0.39, 0.40-0.59, 0.60-0.79, 0.80-1.00.

Classification of Normal vs Abnormal

HHG vs HE	P1	P2	P3	P4	P5	P6	P7	P8	P9	P10
TP	22	23	23	21	23	19	23	23	22	21
TN	3	4	5	7	5	6	3	6	6	7
FP	1	0	1	0	1	0	2	0	1	0
FN	0	2	0	1	0	1	0	0	0	0
Sensitivity	100%	92%	100%	95%	100%	95%	100%	100%	100%	100%
	(22/22)	(23/25)	(23/23)	(21/22)	(23/23)	(19/20)	(23/23)	(23/23)	(22/22)	(21/21)
	[85%-100%]	[74%-99%]	[85%-100%]	[77%-100%]	[85%-100%]	[75%-100%]	[85%-100%]	[85%-100%]	[85%-100%]	[84%-100%]
Specificity	75%	100%	83%	100%	83%	100%	60%	100%	86%	100%
	(3/4)	(4/4)	(5/6)	(7/7)	(5/6)	(6/6)	(3/5)	(6/6)	(6/7)	(7/7)
	[19%-99%]	[40%-100%]	[36%-100%]	[59%-100%]	[36%-100%]	[54%-100%]	[15%-95%]	[54%-100%]	[42%-100%]	[59%-100%]
PPV	96%	100%	96%	100%	96%	100%	92%	100%	96%	100%
	(22/23)	(23/23)	(23/24)	(21/21)	(23/24)	(19/19)	(23/25)	(23/23)	(22/23)	(21/21)
	[80%-99%]	[85%-100%]	[79%-99%]	[84%-100%]	[79%-99%]	[82%-100%]	[80%-97%]	[85%-100%]	[78%-99%]	[84%-100%]
NPV	100%	67%	100%	88%	100%	86%	100%	100%	100%	100%
	(3/3)	(4/6)	(5/5)	(7/8)	(5/5)	(6/7)	(3/3)	(6/6)	(6/6)	(7/7)
	[29%-100%]	[35%-88%]	[48%-100%]	[51%-98%]	[48%-100%]	[47%-98%]	[29%-100%]	[54%-100%]	[54%-100%]	[59%-100%]
Accuracy	96%	93%	97%	97%	97%	96%	93%	100%	97%	100%
	(25/26)	(27/29)	(28/29)	(28/29)	(28/29)	(25/26)	(26/28)	(29/29)	(28/29)	(28/28)
	[80%-100%]	[77%-99%]	[82%-100%]	[82%-100%]	[82%-100%]	[80%-100%]	[76%-99%]	[88%-100%]	[82%-100%]	[88%-100%]
Cohen's kappa	0,84	0,76	0,89	0,91	0,89	0,90	0,71	1,00	0,90	1,00
	[0,52-1]	[0,44-1]	[0,67-1]	[0,74-1]	[0,67-1]	[0,7-1]	[0,33-1]	[1-1]	[0,71-1]	[1-1]

S5: Pathologists' assessment of non-tumor and tumor tissue

Supplementary Data 5 Assessment of normal (N), tumor (T), reactive (R), inflammatory (I), necrotic (NC), other abnormal tissue (O) and abnormal but uncertain which type (A?). Note the difference in meaning between '?' and 'A?', in the former one the pathologist could not classify the fragment at all, while in the latter one the pathologist classified the fragment as abnormal but was not sure about the type(s) of abnormal tissue. Colors are used for a better visualization: normal (green), abnormal non-tumor (orange), tumor (red), and uncertain (white). The color of the 'case' column represents the macroscopic evaluation, i.e. whether the tissue was provided as tumor sample (red) or normal sample (green). Each case consists of two rows, an HHG part (upper row) and an H&E part (lower row). The columns P1-P10 represent the ten pathologists in random order. The consensus was determined as the majority opinion (≥ 6 pathologists in agreement regarding tumor or non-tumor). In two cases there was no clear majority opinion (case 10 HHG, case 16 HHG) and the consensus was classified as abnormal-non-tumor (orange). The consensus was used to calculate the diagnostic characteristics for the distinction between non-tumor (green & orange) and tumor (red).

Case		Pathologists										Consensus
		P1	P2	P3	P4	P5	P6	P7	P8	P9	P10	
01 N	HHG	T, NC, I	N	N	N	N	N	N	N	N	N	
	H&E	N	N	N	N	N	N	N	N	N	N	
02 WT	HHG	T	T	T	T, NC	T, NC	A?	T, A?	T	T	R, NC	
	H&E	T	T	T	T	T, I, O	T	T	T	T	T, R	
03 WT	HHG	T	T	T	T, NC	T	?	T	T	T	R	
	H&E	T	T	T	T	T	T	T	T	T	T, R	
04 WT	HHG	T, I	T	T	T, R, NC	T, R, NC	T	T	T	T	R, O	
	H&E	T	T	T	T, R, I	T, I	T, NC	T	T	T	T	
05 WT	HHG	T, NC, I	T	T	T, R, NC, I	T, R, NC	T, NC	O	T	T	T, O	
	H&E	T	T, R	T	T	T, I	T, NC	T, R, NC	T	T	R	
06 WT	HHG	T	T	T	T	T	T	R, I, O	T	T, R	R, NC, I, O	
	H&E	T	T	T	T	T, R, I	T	T, I	T	T, R	R, I, O	
07 RCC	HHG	T, I	T, R	NC	N	T	T, NC	T, R, I	A?	T, R	NC	
	H&E	T	T, R	T	T	T, I	T	T, R, I	T	T, R	T	
08 WT	HHG	T, R, NC	O	R, NC	R	T	T	R, NC, I	A?	R, I	R, I, O	
	H&E	NC	NC, O	R, NC	R	R, I	R, NC	R, NC, I	NC	R, NC, I	R, NC, I	
09 N	HHG	?	A?	R	N	T	N	O	I, A?	R	N	
	H&E	N	O	R, I	N	T	R	N	I	N	N	
10 WT	HHG	T, NC, I	NC	NC	T, R, NC, I	T, R	T, NC	R, NC	R, NC	R, I	T, NC, I	
	H&E	T, NC	NC	NC	T, NC	T, R, I	T, NC	R, NC	NC	R, NC	NC, I	
11 WT	HHG	T, NC, I	A?	T	T	T, R	T	A?	T	T, R	T, R	
	H&E	T	T	T	T	T, I	T	T	T	T, R	T, R	
12 WT	HHG	T	T	T	T	T, R	?	T	T	T, R, NC	T	
	H&E	T	T, A?	T	T	T, I	A?	T	T, A?	T	T, R	
13 WT	HHG	T, NC	T, NC	NC, I	T, R, NC	T, O	T	T, NC	NC, I	T, R	R, NC, I	
	H&E	T	T, R, NC, I	T	T, R, NC, I	T, R, I	T, R, I	T, R, NC	T, NC, I	T, R, I	T, R, I, O	
14 N	HHG	?	N	N	N	N	N	N	N	N	N	
	H&E	N	N	N	N	N	N	N	N	N	N	
15 WT	HHG	T	T, R, NC, I	T	T, NC	T, R, I	T, R, I	T, R	T, NC, I	T, R	R, A?	
	H&E	T	T, R	T	T	T, I	T, R	T, R	T, R, I	T, R	T	
16 WT	HHG	T, NC, I	R, I, O	T	T, NC	T	?	A?	R, NC, I	R, O	?	
	H&E	T	R, I	R	R, I	T, R, I	R, NC, I	A?	R, NC, I	R, I	?	
17 WT	HHG	T	T	T	T	T	T, NC	T, R	T	T	T	
	H&E	T	T	T	T	T	T	T, R	T	T	T, R	
18 WT	HHG	T	T, NC	T	T	R	T, NC	T, R, NC, I	A?	T, R, NC, I	T, R, NC, I	
	H&E	T	T	T	T	T	T, R	O	A?	T	R, O	
19 WT	HHG	T	T, R, A?	T	T, R	T	T, R, NC	A?	T, NC, I	T, R	R, NC, O	
	H&E	T	T, O	T	T, R	T, R, I	T, R, NC	T, R	T, NC, I	T, R	T, R, NC, O	
20 WT	HHG	T	T, R	T	T, R	T, R	T, R	R, I	T	T, R	R, NC, I	
	H&E	T	T, R	T	T	T, R, I	T, R, I	T, R	T	T, R	T, R, NC	
21 WT	HHG	T	T	T	T, NC	T	T, R	T, R, NC, I	T	T, R	R, NC, I	
	H&E	T	T, R, I	T	T	T, R, I	T, R, I	T, R	T	T, R	T, R, NC, I	
22 RCC	HHG	T, I	T, R, NC, O	T	T, NC	T	T	O	NC, I, A?	T, R	R, NC, I	
	H&E	T, I	T, R, NC	T	T	T	T	T, R, NC, I	T	T	T	
23 CMN	HHG	T	T	T	T	T	T	T, R, I	T	T	T	
	H&E	T	T	T	T	T	T	T, R, I	T	T	T	
24 N	HHG	N	N	N	N	N	N	N	N	N	N	
	H&E	N	N	N	N	N	N	N	N	N	N	
25 WT	HHG	T, NC, I	T, R, NC	R, NC, I	T, R	T, R	T, NC	A?	T, NC, I	T, R, NC	R, NC, I	
	H&E	NC	T, R, NC	NC	NC	R, NC, I	T, NC	NC	NC, I	T, R, NC	R, NC, O	
26 N	HHG	?	N	T	N	N	N	?	N	N	N	
	H&E	N	R, I, O	N	N	N	N	N	N	N	N	
27 N	HHG	N	N	N	N	N	N	R, I	N	N	N	
	H&E	N	R, I, A?	N	N	N	N	O	N	N	N	
28 N	HHG	N	N	N	N	T	N	A?	N	N	N	
	H&E	N	N	N	N	N	N	N	N	N	N	
29 WT	HHG	T	T, NC	T	T	T	T	T, R	T	T, R	T	
	H&E	T	T	T	T	T	T, R	T	T	T	T	

Legenda	
N	Normal: Non-Tumor
	Abnormal: Non-Tumor
T	Abnormal: Tumor
?	Uncertain
**Other abbreviations abnormal tissue:**	
R	Reactive
NC	Necrotic
I	Inflammatory
O	Other
A?	Abnormal, uncertain which type

S6: Statistical analysis of non-tumor and tumor tissue

Supplementary Data 6 Statistical analysis (sensitivity, specificity, PPV, NPV, overall agreement and Cohen's kappa) based on the HHGM assessment of each pathologist compared to their own H&E assessment, regarding non-tumor versus tumor. Calculations are given in parenthesis, 95% confidence intervals are given in square brackets. Colors are used for a better visualization of the percentages: ●0%-24%, ●25%-49%, ●50%-74%, ●75%-100%, and for the kappa values: <0.00, 0.00-0.19, 0.20-0.39, 0.40-0.59, 0.60-0.79, 0.80-1.00.

Classification of Non-Tumor vs Tumor

HHG vs HE	P1	P2	P3	P4	P5	P6	P7	P8	P9	P10
**TP**	20	18	16	18	20	17	11	14	19	5
**TN**	3	10	9	8	5	7	10	11	10	10
**FP**	3	0	2	2	3	1	1	1	0	3
**FN**	0	1	2	1	1	1	6	3	0	10
**Sensitivity**	100%	95%	89%	95%	95%	94%	65%	82%	100%	33%
	(20/20)	(18/19)	(16/18)	(18/19)	(20/21)	(17/18)	(11/17)	(14/17)	(19/19)	(5/15)
	[83%-100%]	[74%-100%]	[65%-99%]	[74%-100%]	[76%-100%]	[73%-100%]	[38%-86%]	[57%-96%]	[82%-100%]	[12%-62%]
**Specificity**	50%	100%	82%	80%	63%	88%	91%	92%	100%	77%
	(3/6)	(10/10)	(9/11)	(8/10)	(5/8)	(7/8)	(10/11)	(11/12)	(10/10)	(10/13)
	[12%-88%]	[69%-100%]	[48%-98%]	[44%-97%]	[24%-91%]	[47%-100%]	[59%-100%]	[62%-100%]	[69%-100%]	[46%-95%]
**PPV**	87%	100%	89%	90%	87%	94%	92%	93%	100%	63%
	(20/23)	(18/18)	(16/18)	(18/20)	(20/23)	(17/18)	(11/12)	(14/15)	(19/19)	(5/8)
	[75%-94%]	[81%-100%]	[69%-97%]	[72%-97%]	[73%-94%]	[73%-99%]	[62%-99%]	[68%-99%]	[82%-100%]	[33%-85%]
**NPV**	100%	91%	82%	89%	83%	88%	63%	79%	100%	50%
	(3/3)	(10/11)	(9/11)	(8/9)	(5/6)	(7/8)	(10/16)	(11/14)	(10/10)	(10/20)
	[29%-100%]	[60%-99%]	[54%-94%]	[54%-98%]	[41%-97%]	[51%-98%]	[46%-77%]	[56%-91%]	[69%-100%]	[39%-61%]
**Accuracy**	88%	97%	86%	90%	86%	92%	75%	86%	100%	54%
	(23/26)	(28/29)	(25/29)	(26/29)	(25/29)	(24/26)	(21/28)	(25/29)	(29/29)	(15/28)
	[70%-98%]	[82%-100%]	[68%-96%]	[73%-98%]	[68%-96%]	[75%-99%]	[55%-89%]	[68%-96%]	[88%-100%]	[34%-72%]
**Cohen's kappa**	0,61	0,93	0,71	0,77	0,63	0,82	0,51	0,72	1,00	0,10
	[0,19-1]	[0,78-1]	[0,44-0,97]	[0,51-1]	[0,29-0,97]	[0,58-1]	[0,2-0,83]	[0,47-0,97]	[1-1]	[-0,26-0,46]

S7: Pathologists' assessment of tumor percentages

Supplementary Data 7 Assessment of the tumor percentages. Colors are used for a better visualization, higher tumor percentages have a darker color. The color of the 'case' column represents the macroscopic evaluation, i.e. whether the tissue was provided as tumor sample (red) or normal sample (green). The columns P1-P10 represent the ten pathologists in random order. The mean and standard deviation (SD) are calculated in the last column. This table was used to exclude the necrotic/reactive cases in the calculation of the diagnostic characteristics for the tumor types and Wilms tumor components. Cases that were <33% tumor, based on the H&E mean tumor percentage, are excluded from this calculation.

Case		Pathologists										Mean ± SD
		P1	P2	P3	P4	P5	P6	P7	P8	P9	P10	
01 N	HHG	80	0	0	0	0	0	0	0	0	0	8±24
	H&E	0	0	0	0	0	0	0	0	0	0	0±0
02 WT	HHG	99	95	80	95	90	0	90	85	90	0	72±37
	H&E	100	95	90	100	100	98	90	98	90	95	96±4
03 WT	HHG	100	99	100	90	100	0	80	95	90	0	75±38
	H&E	100	100	100	100	100	98	100	100	90	90	98±4
04 WT	HHG	100	70	100	20	20	75	40	80	90	0	60±35
	H&E	100	100	100	70	100	65	40	100	90	80	85±20
05 WT	HHG	70	85	20	30	40	40	0	70	80	80	52±28
	H&E	100	70	90	100	100	80	20	80	80	0	72±33
06 WT	HHG	100	95	90	100	100	85	0	85	80	0	74±37
	H&E	100	100	100	100	80	90	95	100	90	0	86±29
07 RCC	HHG	20	50	0	0	100	80	80	0	30	0	36±37
	H&E	100	95	100	100	100	98	80	100	95	100	97±6
08 WT	HHG	90	0	0	0	100	85	0	0	0	0	28±42
	H&E	0	0	0	0	0	0	0	0	0	0	0±0
09 N	HHG	0	0	0	0	100	0	0	0	0	0	10±30
	H&E	0	0	0	0	100	0	0	0	0	0	10±30
10 WT	HHG	30	0	0	10	40	10	0	0	0	40	13±16
	H&E	5	0	0	1	5	2	0	0	0	0	1±2
11 WT	HHG	90	0	70	90	90	95	0	70	80	90	68±35
	H&E	100	100	100	100	100	95	100	100	95	95	99±2
12 WT	HHG	100	90	100	100	90	0	80	85	70	85	80±28
	H&E	100	100	100	100	100	0	90	85	90	85	85±29
13 WT	HHG	20	5	0	70	20	99	40	0	50	0	30±32
	H&E	100	30	40	10	40	55	60	10	40	70	46±26
14 N	HHG	0	0	0	0	0	0	0	0	0	0	0±0
	H&E	0	0	0	0	0	0	0	0	0	0	0±0
15 WT	HHG	100	40	80	90	90	70	60	10	80	0	62±33
	H&E	100	90	100	100	100	50	80	80	90	80	87±15
16 WT	HHG	90	0	70	90	90	0	0	0	0	0	34±42
	H&E	100	0	0	0	80	0	0	0	0	0	18±36
17 WT	HHG	100	100	100	100	100	95	90	90	90	85	95±5
	H&E	100	100	100	100	100	98	90	99	100	95	98±3
18 WT	HHG	100	90	20	80	0	75	30	0	20	50	47±36
	H&E	100	100	100	100	100	90	0	0	90	0	68±45
19 WT	HHG	100	?	100	80	100	5	0	60	80	0	58±42
	H&E	100	60	100	60	90	55	60	75	60	95	76±18
20 WT	HHG	100	80	100	80	90	65	0	85	90	0	69±36
	H&E	100	90	100	100	90	65	50	95	90	80	86±16
21 WT	HHG	100	80	100	90	100	85	20	90	80	0	75±33
	H&E	100	80	100	100	90	55	90	90	90	5	80±28
22 RCC	HHG	90	70	100	80	100	100	0	0	80	0	62±42
	H&E	90	80	100	100	100	100	40	90	90	95	89±17
23 CMN	HHG	100	99	100	100	100	100	90	100	90	95	97±4
	H&E	100	100	100	100	100	100	90	100	90	99	98±4
24 N	HHG	0	0	0	0	0	0	0	0	0	0	0±0
	H&E	0	0	0	0	0	0	0	0	0	0	0±0
25 WT	HHG	90	50	0	40	90	85	0	25	10	0	39±36
	H&E	0	1	0	0	0	1	0	0	1	0	0±0
26 N	HHG	0	0	100	0	0	0	0	0	0	0	10±30
	H&E	0	0	0	0	0	0	0	0	0	0	0±0
27 N	HHG	0	0	0	0	0	0	0	0	0	0	0±0
	H&E	0	0	0	0	0	0	0	0	0	0	0±0
28 N	HHG	0	0	0	0	100	0	0	0	0	0	10±30
	H&E	0	0	0	0	0	0	0	0	0	0	0±0
29 WT	HHG	100	95	80	100	100	100	90	85	90	85	93±7
	H&E	100	100	100	100	100	98	90	100	100	99	99±3

S8: Pathologists' assessment of tumor diagnosis

Supplementary Data 8 Assessment of the tumor diagnosis. Cases that contained less than 33% tumor are faded. Comments are shown if relevant. The case color represents the gold standard diagnosis. The consensus is determined as the majority opinion, including the comments where only one diagnosis was given. If there was no clear majority opinion, the consensus is classified as 'T'.

Case		Pathologists P1	P2	P3	P4	P5	P6	P7	P8	P9	P10	Consensus
01 N	HHG	WT										N
	H&E											N
02 WT	HHG	WT	WT	WT	WT	WT		Epithelial	RT/WT/?	WT/RCC		WT
	H&E	WT	WT	WT	WT	WT	WT	WT	WT	WT	WT	WT
03 WT	HHG	WT	WT	WT	WT	WT		WT	SC/WT?	T?		WT
	H&E	WT?	WT	WT	WT	WT	WT meta?	RCC/CCC	SC/WT/HB?	WT	WT	WT
04 WT	HHG	WT	WT	WT	WT	WT	T?	WT	WT	WT		WT
	H&E	WT	WT	WT	WT	WT	WT	WT	WT	WT	CMN	WT
05 WT	HHG	WT	WT	WT	WT	WT	WT		WT/CMN	CMN	CMN	WT
	H&E	WT	WT	WT	WT	WT	WT	WT	WT	WT		WT
06 WT	HHG	WT	WT	RT	WT	WT	T?		WT	WT		WT
	H&E	WT	WT	WT	WT	WT	WT	WT	WT	WT		WT
07 RCC	HHG	WT	T?			WT	RCC	Meta		RCC		T
	H&E	RCC	RCC	RCC	RCC	RCC	RCC	Meta C	RCC	RCC	RCC	RCC
08 WT	HHG	WT				GNB	CCSK					
	H&E											
09 N	HHG					Dys						
	H&E					Dys/CMN						
10 WT	HHG	RT			WT	WT	WT				WT?	
	H&E	WT			WT	WT	WT					
11 WT	HHG	WT		WT	WT	WT	WT		WT	WT	WT	WT
	H&E	WT	WT	WT	WT	WT	T?	CMN	WT	WT	WT	WT
12 WT	HHG	WT	WT	RCC	WT	WT		WT	WT	WT	RCC?	WT
	H&E	WT	WT	RCC	WT	WT		WT	WT	WT	WT	WT
13 WT	HHG	T?	T?		WT	WT	RCC	WT+chemo?		WT		WT
	H&E	WT	WT	WT	WT	WT	WT	WT	WT	WT	WT	WT
14 N	HHG											N
	H&E											N
15 WT	HHG	WT	WT	WT	WT	Fibrosis	WT	WT	T?	WT		WT
	H&E	WT	WT	WT	WT	WT	WT	WT	WT	WT	WT	WT
16 WT	HHG	WT		WT	WT	WT						
	H&E	T?				WT						
17 WT	HHG	WT?	WT	WT	WT	WT	RT	WT-chemo?	RT/CMN/WT	WT	WT/RT	WT
	H&E	WT	WT	WT	WT	WT	WT	WT	WT	WT	WT	WT
18 WT	HHG	Anaplasia/C	WT	RCC	RCC		WT	WT		WT	WT	WT
	H&E	WT/MA/RCC	WT	RCC	WT	WT/MA	RCC			MA		T
19 WT	HHG	WT	WT	WT	WT	WT	WT		WT	WT		WT
	H&E	WT	WT	WT	WT	WT	WT	WT	WT	WT	WT	WT
20 WT	HHG	WT	WT	WT	WT	WT	WT		WT	WT		WT
	H&E	WT	WT	WT	WT	WT	WT	WT	WT	WT	WT	WT
21 WT	HHG	WT	WT	WT	WT	CMN	WT	WT/CL?	WT	WT		WT
	H&E	WT	WT	WT	WT	WT	WT	WT	WT	WT	WT	WT
22 RCC	HHG	RCC	T?	RCC	RCC	RCC	RCC			RCC		RCC
	H&E	RCC	RCC	RCC	RCC	RCC	RCC	PET	RCC	RCC	RCC	RCC
23 CMN	HHG	CMN	CMN	CMN	CMN	CMN	CMN	CMN	CMN	CMN	CMN	CMN
	H&E	CMN	CMN	CMN	CMN	WT	CMN	CMN	CMN	CMN	CMN	CMN
24 N	HHG											N
	H&E											N
25 WT	HHG	T?	WT		WT	WT	WT		WT?	WT		WT
	H&E		WT				WT			WT		
26 N	HHG			WT								N
	H&E											N
27 N	HHG											N
	H&E											N
28 N	HHG					RT						N
	H&E											N
29 WT	HHG	WT	WT	WT	WT	WT	WT	WT	RT/WT	WT	WT	WT
	H&E	WT	WT	WT	WT	WT	WT	WT	WT	WT	WT	WT

Legenda	
	No tumor
WT	Wilms tumor
RCC	Renal cell carcinoma
CMN	Congenital mesoblastic nephroma
RT	Rhabdoid tumor
	Other tumor
	Tumor, uncertain which type
**Other abbreviations**	
SC	Small-medium cell
meta	Metastasis
CCC	Clear cell carcinoma
HB	Hepatoblastoma
C	Carcinoma
GNB	Ganglioneuroblastoma
CCSK	Clear cell sarcoma of the kidney
Dys	Renal dysplasia
MA	Metanephric adenoma
CL	Congenital lesion
PET	Papillary epithelial tumor
WT+chemo	Wilms tumor after chemotherapy
WT-chemo	Wilms tumor before chemotherapy

S9: Pathologists' assessment of Wilms tumor components

Supplementary Data 9 Assessment of Wilms tumor components. Cases that were not Wilms tumors or contained less than 33% tumor are faded. Percentages are given in the order of blastema, epithelium, stroma. Colors represent the major (>66%) component: blastema (green), epithelium (orange), stroma (red), mixed (grey) if no component is above 66%, and undeterminable (black). Percentages that did not sum up to 100% were normalized for this classification. Anaplasia (A) and rhabdomyoblastic differentiation (RD) are indicated in bold letters. The consensus is determined as the majority opinion. If there was no clear majority opinion, the consensus is classified as mixed. It should be noted that even if the colors are different, the percentages can be close together. Especially cases 11, 12 and 29 had a high interobserver variability, even on histology.

Case		Pathologists										Consensus
		P1	P2	P3	P4	P5	P6	P7	P8	P9	P10	
01 N	HHG	20, 70, 10										
	H&E											
02 WT	HHG	10, 90, 0	100, 0, 0	90, 10, 0	100, 0, 0	100, 0, 0						Blastemal
	H&E	100, 0, 0	100, 0, 0	90, 10, 0	100, 0, 0	70, 20, 10	100, 0, 0	100, 0, 0	100, 0, 0	98, 1, 1	95, 0, 5	Blastemal
03 WT	HHG	100, 0, 0	100, 0, 0	100, 0, 0	100, 0, 0	100, 0, 0 **A**		0, 80, 0				Blastemal
	H&E		95, 5, 0	100, 0, 0	100, 0, 0	80, 20, 0				40, 60, 0	20, 75, 5	Blastemal
04 WT	HHG	20, 0, 80 **RD**	15, 5, 80	20, 10, 70 **RD**	20, 10, 70 **RD**	60, 30, 10		0, 80, 0	0, 0, 75 **RD**	2, 40, 60		Stromal
	H&E	20, 10, 70 **RD**	10, 10, 80	20, 10, 70 **RD**	5, 15, 80 **RD**	10, 20, 70 **RD**	40, 10, 50 **RD**	30, 30, 40	5, 10, 85	0, 20, 80 **RD**		Stromal
05 WT	HHG	0, 20, 80	15, 20, 65	50, 50, 0	10, 20, 70	30, 70, 0	33, 33, 33 **RD**					Mixed
	H&E	0, 30, 70	2, 38, 60	0, 40, 60	0, 10, 90	0, 20, 80	30, 30, 40	0, 40, 60		0, 10, 90		Mixed
06 WT	HHG	0, 10, 90 **RD**	5, 10, 85		0, 0, 100 **RD**	0, 20, 80 **RD**			0, 0, 95 **RD**	0, 10, 80 **RD**		Stromal
	H&E	0, 0, 100 **RD**	0, 0, 100	0, 0, 100 **RD**	0, 0, 100 **RD**	0, 0, 100 **RD**	0, 0, 100 **RD**	0, 0, 100 **RD**	0, 0, 100 **RD**	0, 0, 100 **RD**		Stromal
07 RCC	HHG	0, 90, 10 **A**				0, 80, 20 **RD**						
	H&E											
08 WT	HHG	0, 0, 90										
	H&E											
09 N	HHG											
	H&E											
10 WT	HHG				0, 20, 80	0, 30, 70 **A RD**	100, 0, 0					
	H&E	0, 100, 0			0, 100, 0	50, 50, 0	40, 60, 0					
11 WT	HHG	100, 0, 0 **A**		20, 80, 0	30, 10, 70	0, 0, 90 **RD**	55, 45, 10 **RD**		0, 60, 40	20, 50, 30	85, 0, 0	Mixed
	H&E	0, 10, 90	0, 50, 50	0, 50, 50	5, 15, 80	0, 20, 80			0, 70, 30	0, 70, 30	10, 90, 0	Mixed
12 WT	HHG	70, 20, 10	20, 80, 0		0, 90, 10	80, 0, 20 **RD**		40, 40, 20	70, 20, 10	30, 60, 10		Mixed
	H&E	0, 20, 80	0, 80, 20		0, 50, 50	0, 20, 80 **RD**		5, 40, 55	0, 85, 15	0, 80, 20	5, 75, 20	Mixed
13 WT	HHG				20, 50, 30	0, 0, 100 **RD**				0, 0, 70 **RD**		Stromal
	H&E	0, 10, 90 **RD**	0, 10, 90	0, 20, 80	0, 10, 90	0, 20, 80	0, 20, 80 **RD**	0, 10, 90	0, 0, 10 **RD**	0, 30, 70 **RD**	0, 5, 95 **RD**	Stromal
14 N	HHG											
	H&E											
15 WT	HHG	40, 30, 30	5, 15, 80	0, 30, 70	5, 20, 75		35, 35, 30 **RD**	40, 30, 30 **A**		5, 25, 70 **RD**		Stromal
	H&E	0, 20, 80 **RD**	10, 10, 80	0, 20, 80	0, 10, 90	0, 20, 80	10, 30, 60	5, 20, 75	0, 30, 70	0, 25, 75 **RD**	1, 9, 90	Stromal
16 WT	HHG	10, 60, 30		10, 20, 70	5, 25, 70	0, 40, 60 **RD**						
	H&E					0, 20, 80						
17 WT	HHG		50, 50, 0	0, 100, 0	50, 50, 0	100, 0, 0				70, 30, 0		Mixed
	H&E	60, 35, 5	55, 40, 5	50, 50, 0	50, 50, 0	50, 40, 20	45, 50, 5	60, 30, 10	70, 30, 0	55, 45, 0	70, 20, 10	Mixed
18 WT	HHG		60, 30, 10 **A**				35, 35, 30 **RD**	10, 80, 10 **A**		30, 70, 0	0, 0, 0	Mixed
	H&E		0, 100, 0		0, 100, 0							Epithelial
19 WT	HHG	0, 0, 90 **RD**	0, 0, 0	0, 0, 100	0, 0, 100	0, 30, 70	15, 50, 35		0, 0, 90 **RD**	10, 15, 75		Stromal
	H&E	0, 10, 90	0, 5, 95	0, 10, 90 **RD**	0, 5, 95 **RD**	0, 10, 90 **RD**	5, 10, 85 **RD**	0, 10, 90	0, 5, 95 **RD**	0, 10, 90	0, 5, 95 **RD**	Stromal
20 WT	HHG	0, 10, 90 **RD**	0, 10, 90	0, 0, 100	0, 20, 80	0, 10, 90 **RD**	30, 30, 40		0, 0, 85 **RD**	0, 20, 80 **RD**		Stromal
	H&E	0, 10, 90 **RD**	0, 20, 80	0, 10, 90 **RD**	0, 10, 90 **RD**	0, 10, 90 **RD**	5, 15, 80 **RD**	0, 30, 70 **RD**	0, 25, 75 **RD**	0, 12, 90	0, 10, 90	Stromal
21 WT	HHG	0, 10, 90 **RD**	5, 10, 85	0, 10, 90	0, 10, 90 **RD**		15, 25, 60		0, 15, 85 **RD**	0, 20, 80 **RD**		Stromal
	H&E	0, 10, 90 **RD**	0, 10, 90	0, 10, 90 **RD**	0, 5, 95 **RD**	0, 10, 90 **RD**	5, 15, 80 **RD**	0, 10, 90 **RD**	0, 10, 90 **RD**	0, 10, 90 **RD**	0, 5, 95 **RD**	Stromal
22 RCC	HHG											
	H&E											
23 CMN	HHG											
	H&E					5, 10, 85						
24 N	HHG											
	H&E											
25 WT	HHG		10, 30, 60		5, 5, 90	20, 50, 30 **RD**	30, 30, 40 **RD**			0, 20, 80		
	H&E		0, 0, 0				0, 100, 0			0, 95, 5		
26 N	HHG			0, 100, 0								
	H&E											
27 N	HHG											
	H&E											
28 N	HHG											
	H&E											
29 WT	HHG	75, 20, 5	20, 80, 0	50, 50, 0	0, 100, 0	90, 0, 10	40, 20, 40	20, 70, 10		90, 10, 0	80, 0, 0	Mixed
	H&E	40, 55, 5 **A**	20, 80, 0 **A**	0, 100, 0	20, 80, 0	30, 70, 0 **A**	20, 80, 0 **A**	85, 10, 5	20, 80, 0	10, 90, 0 **A**	15, 80, 5 **A**	Epithelial

S10: Degree of certainty and correspondence between HHGM and H&E

Supplementary Data 10 Degree of certainty per question and correspondence between HHGM and H&E. Dark green = very certain answer or very good correspondence. Light green = certain answer or good correspondence. Yellow = neutral answer/correspondence. Orange = uncertain answer or bad correspondence. Red = very uncertain answer or very bad correspondence. For each case, the top row is regarding HHG, and the bottom row is regarding H&E. For the correspondence, the HHGM answer ('x') should be empty, but there was probably some misunderstanding in this question.

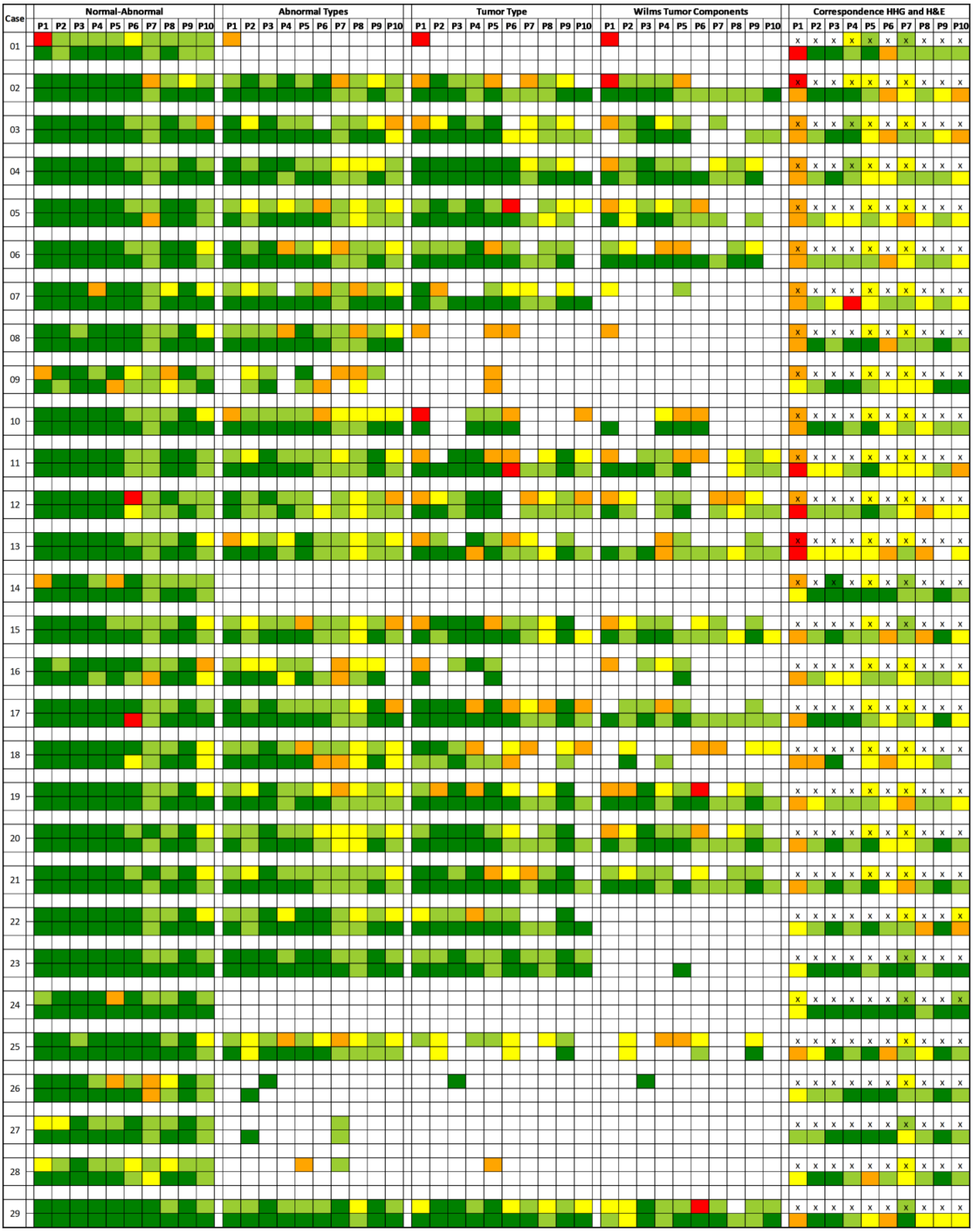

Supplement: Supplementary file 1 [file cancers-17-01693-s001.zip › cancers-3575470-supplementary.pdf]
